# Efficacy and Safety of Orally Administered Acotiamide Extended-Release Tablets Among Functional Dyspepsia-Postprandial Distress Syndrome Patients: A Randomized, Double-Blind, Multicenter Study

**DOI:** 10.7759/cureus.14361

**Published:** 2021-04-08

**Authors:** Shubhadeep D Sinha, Saroj K Sinha, Leela Talluri, Ramesh K Bhashyakarla, Umadevi Malladi, Rupal V Dosi, Mukesh K Jain, Sreenivasa Chary, Mohan Reddy, Pankaj Thakur

**Affiliations:** 1 Clinical Development and Medical Affairs, Hetero Labs Limited, Hyderabad, IND; 2 Gastroenterology, Postgraduate Institute of Medical Education and Research, Chandigarh, IND; 3 Gastroenterology, Osmania General Hospital, Hyderabad, IND; 4 Gastroenterology, Gandhi Hospital, Secunderabad, IND; 5 Internal Medicine, Sir Sayajirao General Hospital and Medical College, Vadodara, IND; 6 Gastroenterology, Sawai Man Singh Hospital, Rajasthan, IND

**Keywords:** functional dyspepsia, rome iv criteria, acotiamide er, prokinetic, epigastric pain syndrome, postprandial distress syndrome, extended release formulation, patient compliance

## Abstract

Background: Acotiamide, is the world's first-in-class, prokinetic drug and world’s first approved treatment for postprandial distress syndrome (PDS) symptoms of functional dyspepsia (FD). An extended-release (ER) formulation of this drug product, developed first-time in the world has been evaluated in phase 3, a comparative trial to explore the efficacy and safety in patients with FD-PDS.

Methods: In this study, 219 patients with FD-PDS aged 18-65 years were randomized (1:1) to receive either acotiamide ER 300 mg once daily or acotiamide 100 mg three times daily for four weeks. The primary efficacy endpoint was responder rates for the overall treatment effect (OTE) at end of week 4. Secondary efficacy endpoints included OTE at each week, elimination rate of postprandial fullness, upper abdominal bloating and early satiation, improvement of individual symptom scores, and quality of life (QoL). The safety endpoints included assessments of treatment-emergent adverse events (TEAEs).

Results: The responder rate for OTE at the end of the four week period, in acotiamide ER 300 mg OD versus acotiamide 100 mg TID group was 92.66% and 94.39% (97.5% CI −8.3,4.8), respectively, in per-protocol (PP) population and 92.66% and 92.73% (97.5% CI −7.0,6.8), respectively, in intent to treat (ITT) population. All other secondary efficacy endpoints, including QoL, were significantly improved with acotiamide ER 300 mg. Both the formulations of acotiamide significantly improved symptom severity and eliminated meal-related symptoms in patients with FD. Adverse events were reported by 7.9% of patients in acotiamide ER 300 mg and 9.2% in acotiamide 100 mg patients; the most common adverse event reported was a headache.

Conclusions: The efficacy and safety of acotiamide ER 300 mg once daily were observed to be comparable to acotiamide immediate release 100 mg thrice daily. A significant improvement in QoL over a four-week treatment period in FD-PDS patients was observed.

## Introduction

Functional dyspepsia (FD) is defined in accordance with the ROME IV criteria [1] as the presence of chronic symptoms of gastroduodenal origin (postprandial fullness [PPF], early satiation [ES], epigastric pain, and burning) without any explanatory organic or metabolic causes. FD is a common morbid condition and has an increased impact on the quality of life (QoL), healthcare, and socioeconomic costs. It is considered a heterogeneous condition in terms of the underlying pathophysiology and therapeutic approach. The symptoms are mostly chronic, occurring at least weekly and over a period of at least six months. In line with Rome IV, FD is subdivided into postprandial distress syndrome (PDS), which is characterized by meal-induced dyspeptic symptoms, and epigastric pain syndrome (EPS) characterized by meal unrelated symptoms (epigastric pain or epigastric burning) [2] and respond differently to therapeutic interventions. FD is often self-medicated by patients with over-the-counter drugs. There are few treatment options with established efficacy to reduce gastric acid secretion and enhance the reduced gut motility.

Patients with EPS benefit from acid secretion inhibitors, whereas patients with PDS benefit from prokinetic drugs such as mosapride and acotiamide. Other treatment options in EPS include H2-blockers and proton pump inhibitors (PPIs). FD significantly impairs health-related QoL, due to symptoms causing emotional distress, problems with food and drink, impaired vitality [3], work productivity and imparts a significant economic burden on patients and the healthcare system. Globally, the prevalence of FD is reported to range between 5% and 11% when using the ROME III criteria [4]. The underlying pathophysiology of FD remains poorly elucidated and being a heterogeneous disorder FD involves multiple pathogenic factors, such as excessive gastric acid secretion, gastric motility disorders, *Helicobacter pylori* infection, psychological factors, and visceral hypersensitivity.

Acotiamide received its first global regulatory approval in Japan in 2013 for the treatment of bloating after meals, epigastric bloating, and early satiety in FD patients. It exerts gastrokinetic activity by enhancement of acetylcholine release via acting as an antagonist on the muscarinic types 1 and 2 autoreceptors in the enteric nervous system and inhibiting acetylcholinesterase activity [5,6]. Acotiamide may act directly on the gut and also indirectly through the brain-gut axis via actions in the central nervous system [7]. It improves upper gastrointestinal motility to relieve abdominal symptoms arising due to impaired GI motility in FD patients. A randomized controlled study showed that acotiamide improves impaired gastric accommodation and delayed gastric emptying [8]. In a phase III study conducted with acotiamide (immediate-release formulation by Hetero), administered 100 mg thrice daily dosage showed a 98% improvement in overall treatment effect (OTE) at week 4. It also showed a statistically significant improved elimination rate of postprandial fullness, upper abdominal bloating, and early satiety at week 4 and also improvements in other parameters such as individual symptom scores, QoL with optimal safety profile (Acotiamide Phase 3 Study Data on File Hetero Labs Limited Submitted to CDSCO 2016). However, the limitation with this product was thrice a day dosing, which had the potential to limit its long-term clinical usage by potentially affecting compliance. Hence, acotiamide extended-release (ER) 300 mg, an ER formulation, was developed to decrease the daily dosing frequency from thrice daily dosing to once daily. In the ER formulation, a suitable combination of diluents at an appropriate ratio had helped in achieving the required drug release pattern, which supported maintaining the required plasma levels of the drug to elicit its pharmacological action for a period of 24 hours.

Therefore, a phase III, randomized double-blinded, multicentric study was conducted to evaluate the performance of acotiamide ER preparation in comparison with the immediate release acotiamide oral formulation.

## Materials and methods

The study results are presented in accordance with the CONSORT statement.

Study design

This phase III, randomized, prospective, multicenter, double-blind, comparative, active-controlled, parallel-group clinical trial was conducted at multispecialty hospitals/centers across India from January 2020 to July 2020. Patients received the study treatment in 1:1 ratio of either acotiamide ER 300 mg and placebo 100 mg in the morning and placebo 100 mg in the evening and night (test group) or acotiamide 100 mg and placebo 300 mg in the morning and acotiamide 100 mg in the evening and night (reference group), i.e., 2 tablets + 1 tablet + 1 tablet, three times a day before meals over a period of four weeks to ensure the treatment balance.

This study was conducted in compliance with the ICH Tripartite guideline regarding Good Clinical Practice (R2, 2018) and Declaration of Helsinki (updated version as amended by the WMA General assembly, Brazil 2013) and New Drugs and Clinical Trial Rules’ 2019 along with subsequent amendments and Indian regulatory laws governing biomedical research in human patients. The study was registered at Clinical Trial Registry-India (CTRI/2019/11/021897) [9] prior to initiation of subject screening. The study was reviewed and approved by individual institutional ethics committees of participating hospitals (clinical trial sites) before its commencement. Written informed consent was obtained from all screened and enrolled subjects prior to study initiation.

Inclusion and exclusion criteria

Patients of either gender aged ≥18 years to ≤65 years, diagnosed as FD-PDS as per the Rome IV classification. Patients with coexisting EPS symptoms were included only if the symptoms causing most distress at the time of study entry were meal-related symptoms (PPF, upper abdominal bloating, or ES). All patients underwent upper abdominal endoscopy at the screening to rule out any abnormalities in the esophagus, duodenum, or stomach. Patients with a history of irritable bowel syndrome, presence of any symptom indicating serious or malignant disease, patients with diabetes mellitus, serious depression or anxiety disorder, hemoglobin levels less than 9 gm/dl, drug or alcohol abuse, biliary tract disease and/or pancreatitis, current human immunodeficiency virus (HIV), hepatitis B (HBV), and C virus (HCV) seropositive and severe abnormality in the electrocardiogram were excluded. Anti-secretory drugs, prokinetics, non-steroidal anti-inflammatory drugs antacids, and antidepressant drugs were not allowed after the baseline period.

Randomization

The study comprised a maximum of one week of screening and baseline period followed by four weeks of the treatment period. During the baseline period, patients were given patient diaries to mark their symptoms with severity on each day to determine the study eligibility. The randomization scheme was generated by permuted block randomization technique by using SAS® (version 9.3 or higher) system software (SAS Institute, Inc., Cary, NC). Eligible patients were assigned unique patient ID and treatment in the ratio of 1:1 to receive either test product or reference product as per the central randomization schedule. This study was a double-blind and double-dummy study wherein the matching placebos were administered to match the frequency of administration.

Efficacy and safety assessments

The primary endpoint was the responder rate based on OTE by using a seven-point Likert scale [10] at the end of the treatment visit. Patients with “extremely improved” or “improved” on the OTE scale were considered as responders. Secondary endpoints included elimination rate of PPF, upper abdominal bloating, and early satiety at the end of treatment visit; OTE by using seven-point Likert scale at each week; The improvement of individual symptom (upper abdominal pain, upper abdominal discomfort, PPF, upper abdominal bloating, ES, excessive belching, nausea, vomiting, and heartburn) score on a severity scale of 0-3 (none, mild, moderate, and severe) at each week and improvement in disease-specific QoL by using Short Form-Nepean Dyspepsia Index questionnaire (SF-NDI) [11]. Treatment-emergent clinical and laboratory adverse events (TEAEs) were assessed as safety endpoints.

Statistical analysis

Efficacy and safety analyses were performed with the intention to treat (ITT) and per-protocol (PP) population. The variables measured on a continuous scale such as age, height, mean, standard deviation, median, and range were compared using a t-test and the proportions like males/females were compared using Fisher’s exact test. The primary endpoint, the responder rate at each visit, was compared by using Fisher’s exact test. The elimination rate at each visit was compared by using Fisher’s exact test. The OTE score, individual symptom score, and SF-NDI scores at each visit were compared between groups by using a t-test. The change from baseline was compared within-group by using paired t-test and between groups by using Analysis of Covariance (ANCOVA). Adverse events were coded using version 19.1 of Medical Dictionary for Regulatory Activities (Med DRA). The incidence of serious adverse events was compared across the treatment groups using Fisher’s exact test. All statistical tests were performed using SAS® (version 9.4 or higher) system software (SAS Institute, Inc.).

## Results

Patient disposition and baseline characteristics

A total of 277 patients were screened, and among them, 219 patients with FD-PDS who met the eligibility criteria as defined by the Rome IV classification were randomized and enrolled in the ratio of 1:1 to receive either acotiamide ER 300 mg OD (109 patients) or acotiamide 100 mg TID (110 patients) before meals. ITT population consisted of 219 (100%) patients (109 (100%) patients in acotiamide ER 300 mg OD and 110 (100%) patients in acotiamide 100 mg TID) and PP population consisted of 216 (98.63%) patients (109 (100%) patients in acotiamide ER 300 mg OD and 107 (97.27%), 3 patients were lost to follow up) patients in acotiamide 100 mg TID. Safety analysis was conducted for 219 patients. The baseline characteristics of the trial population were similar between the acotiamide ER 300 mg and acotiamide 100 mg groups (Table 1).

**Table 1:** Demographic and baseline characteristics of the patients Data are shown as mean ± SD or n (%); *p-values are obtained by performing Fisher’s exact test; **p-values are obtained by performing a t-test.

Characteristics	Acotiamide ER 300 mg OD N=109	Acotiamide 100 mg TID N=110	P-value
Gender			
Male	73 (66.36)	71 (65.14)	
Female	36 (32.73)	39 (35.78)	0.7762^*^
Age (years)	39.6 ± 11.3	38.0 ± 10.7	0.2645^**^
Height (cm)	162.8 ± 7.7	163.3 ± 8.4	0.6699^**^
Weight (kg)	63.1 ± 7.9	64.2 ± 9.6	0.3701^**^
BMI (kg/m^2^)	23.8 ± 2.9	24.1 ± 3.1	0.5988^**^
Ethnicity			
Hispanic/Latino	-	2 (1.83)	0.4977^*^
Not Hispanic/Latino	109 (99.09)	108 (99.08)	
Race			
Asian	109 (100%)	110 (100%)	-

Primary efficacy endpoint

The responder rate based on the OTE at the end of the treatment visit (week 4) was 92.66% and 94.39% (PP population) and 92.66% and 92.73% (ITT population) in acotiamide ER 300 mg OD and acotiamide 100 mg TID, respectively. A significant difference became apparent from week 2 in both the treatment groups. The difference in proportion between groups was comparable [−1.7% (−8.3, 4.8); P = 0.7835) and −0.1% (−7.0, 6.8); P = 1.0000)] in PP and ITT population, respectively (Table 2).

**Table 2:** Efficacy endpoints at week 4 per-protocol population and intent to treat population PP: per-protocol, OTE: overall treatment effect, PPF: postprandial fullness, ES: early satiation.

Endpoint	Acotiamide ER 300 mg OD N=109 n (%)	Acotiamide 100 mg TID N=110 n (%)	Difference (95% CI)	Acotiamide ER 300 mg OD vs. acotiamide 100 mg TID (p-value)
(A) PP population				
Primary endpoint responder rate by using OTE	92.66	94.39	−1.7 (−8.3, 4.8)	0.7835
Secondary endpoints elimination rate PPF, upper abdominal bloating, ES	38.53	37.38	−1.1 (−14.1, 11.8)	0.8892
(B) Intent to treat population				
Primary endpoint responder rate by using OTE	101 (92.66)	102 (92.73)	−0.1 (−7.0,6.8)	1.0000
Secondary endpoints elimination rate PPF, upper abdominal bloating, ES	38.53	36.36	−2.2 (−15.0,10.6)	0.7810

Secondary efficacy endpoints

Overall Treatment Effect at Weeks 1 to 4

The responder rates in PP population in acotiamide ER 300 mg OD and acotiamide 100 mg TID groups were 24.77% vs. 14.02% [(0.3, 21.2); p= 0.0583)], 46.79 % vs. 45.79% [(−12.3, 14.3); p= 0.8924)], 81.65% vs. 79.44% [(−8.3, 12.8); p= 0.7327)] and 92.66% vs. 94.39% [(−8.3, 4.8); p= 0.7835)] at weeks 1, 2, 3, and 4, respectively. Responder rates in ITT population in acotiamide ER 300 mg OD and acotiamide 100 mg TID groups were 24.77 % vs. 14.55% [(−0.2, 20.7); p=0.0629)], 46.79 % vs. 45.45% [(−11.9, 14.5); p=0.8925)], 81.65% vs. 78.18% [(−7.1, 14.1), p=0.6134)] and 92.66% vs. 92.73% (95% CI is −7.0, 6.8; p=1.0000)] at weeks 1, 2, 3, and 4, respectively.

Elimination Rate

The elimination rate of PPF, upper abdominal bloating and early satiety at week 4 in acotiamide ER 300 mg OD and acotiamide 100 mg TID groups were 2.75% vs. 3.74 % [(−3.7, 5.7); p=0.7201)], 3.67% vs. 7.48% [(−2.3, 9.9); p=0.2498], 38.53% vs. 37.38% [(−14.1, 11.8); p=0.8892)] in PP population and 2.75% vs. 3.64% [(−3.8, 8.8); p=1.000)], 3.67% vs. 7.27% [(−2.4, 9.6); p=0.3741)] and 38.53% vs. 36.36% [(−15.0, 10.6); p=0.7810)] in ITT population, respectively. The elimination rates were comparable between both the test and reference groups (Table 2).

The Improvement of Individual Symptom Scores

The treatment of acotiamide ER 300 mg OD and acotiamide 100 mg TID showed significant improvement rates in individual symptom severity on a severity scale of 0-3 (none, mild, moderate, and severe) at each week. There is no significant difference observed for the difference in improvement rates in individual symptom severity between both the groups (Table 3).

**Table 3:** Individual Symptom Severity Score - change from baseline to week 4 in ITT and PP population N: total number of subjects, ITT: intent to treat, PP: per-protocol, PPF: postprandial fullness, ES: early satiation.

Parameter	PP population				ITT population			
	Acotiamide ER 300 mg OD (N=109) mean ± SD	Acotiamide 100 mg TID (N=107) mean ± SD	Acotiamide ER 300 mg OD vs. acotiamide 100 mg TID		Acotiamide ER 300 mg OD N=109 mean ± SD	Acotiamide 100 mg TID mean ± SD	Acotiamide ER 300 mg OD vs. acotiamide 100 mg TID	
			Mean difference (95%CI)	P-value			Mean difference (95%CI)	P-value
Upper abdominal pain	0.4 ± 0.60	0.4 ± 0.63	−0.02 (−0.2, 0.1)	0.7860	0.4 ± 0.63	0.5 ± 0.74	−0.01 (−0.2, 0.2)	0.9498
Upper abdominal discomfort	0.4 ± 0.63	0.5 ± 0.69	−0.06 (−0.3, −0.2)	0.1210	0.4 ± 0.60	0.5 ± 0.66	−0.08 (−0.1, 0.3)	0.3630
PPF	0.6 ± 0.66	0.7 ± 0.73	0.11 (−0.1, 0.3)	0.2366	0.6 ± 0.66	0.7 ± 0.80	−0.16 (−0.0, 0.4)	0.0911
Upper abdominal bloating	0.5 ± 0.62	0.5 ± 0.74	0.04 (−0.1, −0.2)	0.6356	0.5 ± 0.44	0.5 ± 0.52	0.08 (−0.1, 0.3)	0.3736
ES	0.5 ± 0.60	0.6± 0.72	0.14 (−0.0, 0.3)	0.1265	0.5 ± 0.60	0.4± 0.62	0.17 (−0.0, 0.3)	0.0667
Excessive belching	0.3 ± 0.55	0.3 ± 0.60	0.06 (−0.1, 0.2)	0.4801	0.3 ± 0.55	0.3 ± 0.39	−0.08 (−0.1, 0.2)	0.2973
Nausea	0.2 ± 0.50	0.2 ± 0.48	0.03 (−0.1, 0.2)	0.6954	0.2± 0.50	0.2 ± 0.48	0.03 (−0.1, 0.2)	0.6373
Vomiting	0.0 ± 0.00	0.0 ± 0.00	0.00 (0.0, 0.0)	0	0.1 ± 0.22	0.1± 0.19	−0.00 (−0.0, 0.0)	0
Heartburn	0.2 ± 0.56	0.2 ± 0.50	−0.03 (−0.2, 0.1)	0.6620	0.0± 0.00	0.0 ± 0.10		

Quality-of-Life Scores

The SF-NDI QoL score was significantly improved in all parameters compared to baseline at week 4. There is no significant difference observed between the groups in SF-NDI parameters (tension (p=0.7876), interference with daily activities (p=0.5340), eating/drinking (p=0.0525), knowledge/control (p=0.5485) and work/study (p=0.5439) in PP population and in ITT population - tension (p=0.4117), interference with daily activities (p=0.2697), eating/drinking (p=0.0137) and knowledge/control (p=0.3573)).The work/study (p=0.7030) parameter was significantly different between groups in the ITT population (Table 4).

**Table 4:** Summary of overall and subscale symptom scores on the Short Form-Nepean Dyspepsia Index questionnaire N: total number of subjects, ITT: intent to treat, PP: per-protocol; P-values are obtained by performing analysis of covariance (ANCOVA).

Change from baseline to week 4 for SF-NDI (score), mean ±SD								
Variable	PP population				ITT population			
	Acotiamide ER 300 mg OD (N=109) mean ± SD	Acotiamide 100 mg TID (N=107) mean ± SD	Acotiamide ER 300 mg OD vs. acotiamide 100 mg TID		Acotiamide ER 300 mg OD (N=109) mean ± SD	Acotiamide 100 mg TID (N=110) mean ± SD	Acotiamide ER 300 mg OD vs. acotiamide 100 mg TID	
			Mean difference (95%CI)	P-value			Mean difference (95%CI)	P-value
Tension	3.3 ± 1.29	3.4 ± 1.29	0.05 (−0.3, 0.4)	0.7876	3.3±1.29	3.5 ± 1.42	0.15 (−0.2, 0.5)	0.4117
Interference with daily activities	3.1 ± 1.29	3.3 ± 1.39	0.11 (−0.2, 0.5)	0.5340	3.1 ±1.29	3.4 ± 1.47	0.20 (−0.2, 0.6)	0.2697
Eating/drinking	3.4 ± 1.29	3.7 ± 1.41	0.32 (−0.0, 0.7)	0.0525	3.4 ± 1.29	3.8 ± 1.60	0.44 (0.1, 0.8)	0.0137
Knowledge/control	2.8 ± 1.18	3.0 ± 1.10	0.08 (−0.2, 0.4)	0.5485	2.8 ± 1.18	3.0 ± 1.13	0.13 (−0.1, 0.4)	0.3573
Work/study	2.8 ± 1.44	2.8 ± 1.59	−0.11 (−0.5, 0.2)	0.5439	2.8 ± 1.44	2.8 ± 1.72	−0.07 (−0.4, 0.3)	0.7030

Safety

Overall, 13 adverse events (AE) were reported in 13 patients in the study. Six (7.9%) patients in acotiamide ER 300 mg and 7 (9.2%) in acotiamide 100 mg reported AEs during the study. The most common AE reported was a headache. All the AEs were mild in severity, unlikely related to the study drug, and recovered without any sequelae. There were no deaths and serious AEs reported in the study (Table 5).

**Table 5:** Overall summary of treatment-emergent adverse events - safety population Adverse events are classified by System Organ Class and Preferred Term as defined by the Medical Dictionary of Regulatory Affairs (MedDRA) v21.0.

System organ class preferred term	Acotiamide ER 300 mg OD% AE	Acotiamide 100 mg TID% AE	Overall % AE
Any treatment-emergent AE	7.9	9.2	8.6
Gastrointestinal disorders	1.3	1.3	1.3
Diarrhea	1.3	1.3	1.3
General disorders and administration site condition	3.9	1.3	2.6
Pain	0	1.3	0.7
Pyrexia	3.9	0 0	2.0
Infections and infestations	1.3	0 0	0.7
Nasopharyngitis	1.3	0 0	0.7
Musculoskeletal and connective tissue disorders	1.3	0 0	0.7
Back pain	1.3	0 0	0.7
Nervous system disorders	0	5.3	2.6
Headache	0	5.3	2.6
Respiratory, thoracic, and mediastinal disorders	0	1.3	0.7
Cough	0	1.3	0.7

## Discussion

Functional dyspepsia is a chronic gastrointestinal disorder; uncontrolled FD affects negatively the QoL [12] and results in frequent medical consultations. Adherence to a therapeutic regimen largely influence chronic symptoms and the recurrence rate of dyspepsia symptoms. Hence, Acotiamide ER 300 mg, an extended-release formulation was developed to decrease the daily dosing frequency from thrice daily dosing to once daily and thereby increase patient compliance. Acotiamide is a prokinetic drug and has been made available in Japan since 2013 for the treatment of this disease. In phase II studies conducted in Japan and Europe, acotiamide exerted gastroprokinetic activity, improved gastric emptying and accommodation, thereby showed beneficial effects for FD symptoms of PPF, upper abdominal bloating, and early satiation (ES) [13,14]. Behera and Sethi [15] reported approximately 93% of patients’ improvement of FD symptoms after four-week administration of acotiamide. Narayanan et al. [16] reported that complete relief or significant improvement from PPF, upper abdominal bloating, and early satiety was achieved by 79.2%, 74.4%, and 77.1% of patients, respectively (P<0.001 for all vs. no/slight improvement) when treated for >28 days or 14-28 days with acotiamide. Matsueda et al. [17] reported the responder rate based on the OTE was 52.2% receiving acotiamide 100 mg TID and 34.8% of patients receiving placebo (p<0.001); at the end of four weeks acotiamide group (P=0.004) showed improvement in all three meal-related FD symptoms. The elimination rate of all three meal-related symptoms (PPF, upper abdominal bloating, and ES) was 15.3% with acotiamide and 9.0% with placebo (p=0.004). 

The study results showed that the dose of acotiamide ER 300 mg once daily had a consistent efficacy (OTE-92.66%); the elimination rate of all three meal-related symptoms (PPF, upper abdominal bloating, and ES) is 38.53%, a significant improvement on all sub-domains of the disease-specific SF-NDI QoL assessment), which were comparable to the comparator, and efficacy data of acotiamide immediate release in reference arm and those reported in the literature. Similar results were reported in a phase III study conducted with acotiamide (immediate-release formulation by Hetero), administered 100 mg thrice daily dosage in India with a responder rate of 98% at the end of week 4. The higher responder rates reported in this study could be due to differences in the study population in terms of race, ethnicity, and food habits of the Indian population.

## Conclusions

Functional dyspepsia-postprandial distress syndrome is highly prevalent and important clinical issue due to its heterogeneity of underlying pathophysiology and a lack of therapy options. It causes personal distress, somatic symptoms, and a high economic burden in society. Another issue is that of a lack of long-acting options to provide day-long relief of symptoms without the need for multiple dosages. This study showed that a novel formulation of acotiamide ER 300 mg dose orally once daily resulted in significant improvement in OTEs and QoL from baseline to end of treatment with optimum safety which is comparable to that of acotiamide immediate-release 100 mg orally thrice daily. The improved efficacy is also likely to be related to a convenient daily dosing schedule from thrice daily to once daily which might improve treatment compliance.
